# Research on the factors influencing tourist loyalty to outdoor music festivals: an application of stimulus-organism-response paradigm

**DOI:** 10.3389/fpsyg.2025.1553211

**Published:** 2025-05-22

**Authors:** Ning Zhu, Haochen Xu, Xi Zhang, Yuhui Lu

**Affiliations:** ^1^School of Architectural Art and Design, LuXun Academy of Fine Arts, Shenyang, China; ^2^Shanghai Academy of Fine Arts, Shanghai University, Shanghai, China; ^3^Faculty of Creative Arts, Universiti Malaya, Kuala Lumpur, Malaysia

**Keywords:** China, S-O-R paradigm, music festival, existential authenticity, experience quality, loyalty

## Abstract

**Introduction:**

Following the COVID-19 pandemic, China's music festival market has rapidly recovered, attracting growing tourist interest. However, research on factors influencing tourist loyalty remains limited.

**Methods:**

This study applies the Stimulus-Organism-Response (S-O-R) framework to examine loyalty formation among festivalgoers. Data were collected via a questionnaire from 673 attendees of the Shenyang Strawberry Music Festival. Structural equation modeling (SEM) was used for analysis.

**Results:**

Perceived value and satisfaction significantly impact loyalty, with satisfaction showing a stronger effect. Existential authenticity and experience quality enhance emotions, perceived value, and satisfaction. Emotions do not directly influence loyalty but have indirect effects via value and satisfaction.

**Discussion:**

Findings suggest that enhancing existential authenticity and experience quality can indirectly build loyalty. The study offers strategic insights for festival organizers seeking to sustain tourist engagement post-pandemic.

## 1 Introduction

Festivals have become a vital resource for tourism development due to their positive impacts on culture, economy, and society (Armbrecht, 2021). Among these, music festivals cater to tourists' recreational needs with their diverse range of musical genres and open atmosphere, while also providing significant economic and market benefits to host locations (Nunes and Birdsall, 2022). In the wake of the COVID-19 pandemic, the Chinese music festival market has rebounded rapidly and emerged as a key driving force behind the economic recovery of destinations (Su et al., 2024). Therefore, a comprehensive understanding of the mechanisms influencing tourist loyalty is crucial for the sustainable post-pandemic recovery of the festival tourism industry (Chaney and Martin, 2017; Chi et al., 2022).

Existing studies examining tourist loyalty have primarily focused on factors such as performance quality, festival atmosphere, and tourist interactions (Ho et al., 2022; Wang et al., 2024; Sisson and Alcorn, 2022). However, these studies generally focus on specific factors, emphasizing individual psychological and behavioral responses, while paying little attention to the multidimensional interactive mechanisms inherent in festival experiences. Although the interplay among tourist emotions, perceived value, and satisfaction have been extensively explored in tourism research (e.g., Wu and Li, 2017; Ahn and Kwon, 2020; Lee et al., 2014), there remains a lack of systematic investigation into how these factors dynamically influence tourist loyalty within the context of festival tourism. In particular, in the context of post-pandemic recovery, the impact of tourists' existential authenticity and quality of experience on the relationships among emotions, perceived value, and loyalty requires further exploration. Existential authenticity refers to the genuine self-state that an individual experiences within a particular context (Yi et al., 2017). In festival tourism, tourists may experience an enhanced sense of existential authenticity due to unique atmospheres, cultural rituals, or community interactions (Hsu et al., 2021). However, existing studies have largely focused on heritage and nature tourism (e.g., Su et al., 2021; Fu, 2019), offer little systematic explanation of the unique formation mechanisms of existential authenticity in festival tourism—such as cultural rituals and a sense of group belonging. In particular, it remains unclear how existential authenticity indirectly influences tourist loyalty through the mediating roles of emotions and perceived value, a mechanism that still warrants in-depth investigation.

This study addresses these gaps by applying the Stimulus-Organism-Response (S-O-R) paradigm to explore how existential authenticity and experience quality in outdoor music festivals influence emotions, perceived value, and satisfaction, and how these factors ultimately drive loyalty. The study answers three key questions: (1) How do existential authenticity and experience quality impact festivalgoers' emotions, perceived value, and satisfaction? (2) How do emotions and perceived value affect satisfaction? (3) How do these factors influence loyalty? This research aims to provide insights into sustainable music tourism in China and offer practical guidance for festival management.

## 2 Literature review

### 2.1 Stimulus-Organism-Response framework (S-O-R)

The S-O-R paradigm is a critical theoretical framework for understanding consumer and tourist behavior (Choi and Kandampully, 2019). This model posits that environmental stimuli drive behavioral responses by influencing individuals' emotional and cognitive states (Kim et al., 2020; Lin et al., 2023). In the context of tourism and festivals, external stimuli (e.g., experience quality, existential authenticity) can ultimately affect tourist behavior (e.g., satisfaction, loyalty) by impacting their emotional and cognitive states (e.g., emotions and perceived value).

The S-O-R model has been widely applied in tourism research (Chen et al., 2022; Qiu et al., 2023; Fakfare et al., 2024), with existing literature indicating that environmental cues in festivals, such as music festivals, can significantly influence tourists' emotional responses, thereby impacting their behavioral choices (Tan et al., 2023). Fang and Liu (2024) suggest that tourists' emotional responses and cognitive states are crucial throughout the festival experience process, as these not only affect overall satisfaction with the festival but also impact revisit intentions and loyalty.

Building upon this foundational understanding, the present study extends the application of the S-O-R framework by focusing specifically on the context of music festivals. Unlike other event or cultural tourism studies, which have utilized the S-O-R framework, this research uniquely explores how existential authenticity and experience quality influence tourists' satisfaction and loyalty in the context of music festivals. This approach is particularly relevant in the post-pandemic context of China, where tourists' emotional and cognitive responses to cultural and social stimuli have shifted significantly (Su et al., 2024). Thus, this study aims to investigate how these external stimuli impact tourist behavior through emotions and perceived value, contributing new insights into festival tourism, particularly in the rapidly evolving cultural landscape of China.

### 2.2 Existential authenticity

Existential authenticity refers to the alignment between one's inner experiences and outward expressions, staying true to one's genuine state of being (Fu, 2019). In the tourism industry, this concept is closely linked to the overall travel experience and the internal feelings of tourists (Atzeni et al., 2022). Through travel, individuals often achieve a state that deeply resonates with their inner needs and values, allowing them to engage with themselves and others in a more natural and authentic manner (Park et al., 2019). Unlike objective authenticity, which concerns the factual assessment of attractions or cultures, existential authenticity focuses on tourists' subjective experiences (Lee et al., 2024). This is particularly evident when individuals find liberation and self-discovery in unfamiliar environments, free from the constraints of daily life (Meng and Luo, 2024).

Existential authenticity can be further divided into two dimensions: interpersonal authenticity and intrapersonal authenticity (Zhao and Li, 2023). Interpersonal authenticity occurs when individuals can genuinely express their inner feelings, thoughts, and values during interactions with others, without being constrained by external expectations, social norms, or the influence of others (Yi et al., 2017). At events like music festivals, participants often find opportunities to present their true selves and form sincere open social connections (Perron-Brault et al., 2020). The unique atmosphere of music festivals offers a sense of “social liberation,” enabling participants to engage more authentically in interactions, free from the role constraints and social pressures of everyday life (Kruger and Saayman, 2019; Chang et al., 2022).

Intrapersonal authenticity reflects an individual's alignment with their true needs, values, and emotions (Wang, 1999). It means being able to understand oneself genuinely, accept one's inner experiences, and act in accordance with this true self without being influenced by external social norms, others' expectations, or self-deception (Hsu et al., 2021). In the post-pandemic era, the immersive environment of music festivals allows urban tourists to confront inauthentic aspects of themselves (Skandalis et al., 2024), leading to emotional experiences that help alleviate stress, restore emotions, and enhance vitality (Kruger and Saayman, 2019; Zhao, 2023). This, in turn, strengthens their awareness of self-identity and inner needs (Lu et al., 2024).

Heritage tourism emphasizes historical authenticity, cultural heritage, and identity—focusing on cultural accumulation and long-term reflection (Domínguez-Quintero et al., 2019), while nature tourism concentrates on self-rejuvenation and introspection within natural environments (Zhang et al., 2024). In contrast, music festivals offer a more immediate, emotionally charged form of authenticity (Skandalis et al., 2024). Unlike heritage or cultural tourism, where objective authenticity (e.g., historical accuracy, cultural representation) is central, music festivals are not judged by the truthfulness of their content but by how they make participants feel—liberated, emotionally present, and connected (Tan et al., 2020). Their highly immersive and spontaneous atmosphere enables festivalgoers to temporarily escape daily roles, engage in personal meaning-making, and experience emotional catharsis (Perron-Brault et al., 2020).

### 2.3 Experience quality

Tourists evaluate the quality of their travel experience by considering the destination's services, environment, and activities (Moon and Han, 2019). While many researchers have attempted to conceptualize experience quality, a universally accepted theory remains elusive (Walls et al., 2011; Jin et al., 2015; Allan, 2016). Music festivals offer a unique atmosphere that immerses participants in an exotic and reality-escaping experience (Tan et al., 2020). Additionally, music festivals present an environment vastly different from everyday life, exposing attendees to rich cultural and artistic expressions that evoke joy and excitement (Brown and Sharpley, 2019). Being away from routine settings also allows participants to engage more deeply, fostering stronger interactions, and enabling freer self-expression (Matheson, 2008).

To characterize the quality of tourist experiences in outdoor music festivals, this study introduces four new variables: escapism, surprise, engagement, and enjoyment. Escapism reflects the individual's pursuit of experiences that offer relief from the pressures, concerns, or dissatisfaction of daily life (Suhartanto et al., 2020). Surprise represents the freshness or uniqueness of an experience, where unexpected stimuli prompt consumers to reassess their expectations and perceptions of the current experience (Jin et al., 2015). Engagement indicates the depth of interaction a consumer has with a product or service when investing time or money, demonstrating their interest, commitment, and contribution (Suhartanto et al., 2020). Finally, enjoyment refers to the pleasure, satisfaction, or happiness derived from an experience or activity (Moon and Han, 2019). These four dimensions collectively reflect both active participation and passive experience, in accordance with Pine and Gilmore (1998) experience economy theory. Research on tourism experience quality (Walls et al., 2011; Jin et al., 2015; Moon and Han, 2019) indicates that tourist experience derives not only from perceptions of facilities and services but also encompasses psychological feelings and emotional responses. In this study, escapism and engagement represent active involvement (active experience), while surprise and enjoyment underscore the pleasure derived from passive perception (passive experience), collectively reflecting the immersive, emotion-evoking, and escapist characteristics of outdoor music festivals (Brown and Sharpley, 2019). These dimensions not only align with the theoretical foundations of tourism experience quality research but also effectively capture the diverse sensations experienced at music festivals.

### 2.4 Tourist emotions

Tourist Emotions are defined as “a state of readiness arising from cognitive evaluations of events or thoughts; accompanied by a phenomenological tone, physiological processes, bodily expressions, and often a tendency toward specific actions to affirm or address the emotion” (Bagozzi et al., 1999). In tourism, interactions between tourists and service personnel allow tourists to assess empathy and responsiveness, generating emotional responses (Wu et al., 2018). By linking emotions to environmental stimuli and experiences, generally classified as positive emotions (e.g., relaxation, joy, excitement, and surprise) and negative emotions (e.g., anger, dissatisfaction, sadness, and fear) (Laros and Steenkamp, 2005; Karagöz and Uysal, 2022).

Studies highlight how existential authenticity in heritage destinations promotes positive emotions. For instance, authentic cultural expressions, historical context, and heritage sites foster deeper emotional resonance with the destination, enhancing tourists' feelings of joy and satisfaction (Domínguez-Quintero et al., 2019). In family heritage tourism, existential authenticity combined with interpersonal interaction evokes emotional experiences, strengthening family cohesion, and inspiring storytelling behaviors (Meng and Luo, 2024). In natural and cultural parks, experience quality positively correlates with tourist emotions, whereas high-quality experiences evoke positive emotions, and lack of quality can trigger negative emotions (Karagöz and Uysal, 2022). Research further indicates that a destination's physical environment, cultural atmosphere, and facility convenience directly impact tourist emotions (Kim, 2022). In festival tourism, an improved perception of experience quality leads to heightened emotions (Sahin and Kiliçlar, 2023). Thus, this study hypothesizes that:

H1. There is a positive relationship between existential authenticity and tourist emotions.

H2. There is a positive relationship between experience quality and tourist emotions.

### 2.5 Perceived value

Perceived Value refers to tourists' assessment of the net value of a product based on the balance between perceived sacrifices and overall benefits (Chen and Chen, 2023). It is regarded as one of the most important concepts for understanding tourist evaluations in the tourism industry (Ahn and Kwon, 2020). Perceived value in tourism can be divided into two dimensions: functional value and emotional value (Kato, 2021). Functional value relates to the quality of products and services and their monetary worth to tourists, while emotional value reflects the feelings and emotions generated by the tourism experience (Lee et al., 2011). The experiential or hedonic aspects of consumption are commonly seen as foundational elements in conceptualizing perceived value (Miao et al., 2022).

Authentic experiences enhance tourists' emotional involvement and satisfaction, thus increasing their overall sense of value for the tourism activity (Zhang et al., 2019). For instance, Wong et al. (2018) reported a positive relationship between perceived authenticity and perceived value in the context of festival tourism. When tourists engage in festival activities, the authentic cultural atmosphere leads them to perceive higher value in the experience. Similarly, in marine tourism, interpersonal and intrapersonal authenticity positively impact tourists' perceived value (Su et al., 2021). Previous research consistently shows a significant relationship between product/service quality and perceived value (Suhartanto et al., 2020). When experience quality is satisfying, tourists perceive greater value relative to the costs paid (Su et al., 2020). Thus, tourist experience quality is considered a predictor of perceived value. However, findings on the relationship between experience quality and perceived value have been mixed, indicating the need for further exploration (Chen et al., 2020; Li and Shang, 2020). Therefore, the following hypotheses are proposed:

H3. There is a positive relationship between existential authenticity and perceived value.

H4. There is a positive relationship between experience quality and perceived value.

### 2.6 Satisfaction

Satisfaction is an evaluative outcome that indicates whether a product or service experience meets or exceeds expectations (Wu and Li, 2017). Tourists' satisfaction relates to the extent to which both general and specific tourism needs are met (Moon and Han, 2019). Konuk (2019) highlights that individuals use personal experiences to assess service quality, which then shapes their satisfaction. In this study, experience satisfaction is defined as tourists' overall satisfaction with their music festival experience, expanding on the concept of service satisfaction to emphasize an all-encompassing post-consumption evaluation (Pakurár et al., 2019).

While objective and constructive authenticity may not significantly influence satisfaction, research by Genc and Gulertekin Genc (2023) finds a positive relationship between existential authenticity and satisfaction. This suggests that even when certain external factors may not directly impact satisfaction, tourists' subjective sense of authenticity still shapes their overall experience. Studies focused on heritage tourism have supported the link between existential authenticity and satisfaction (Lee et al., 2016), with limited research exploring this relationship in festival tourism.

Research has also extensively examined the relationship between experience quality and satisfaction across various tourism settings, including nature tourism (Moon and Han, 2019), festival tourism (Tanford, 2017), and rural tourism (Chi and Han, 2021). Findings indicate that quality in specific tourism settings has a significant impact on satisfaction. For instance, in music festivals, variables such as educational, entertainment, and aesthetic value are significant predictors of satisfaction (Aşan et al., 2020). Additionally, Tan et al. (2020) report that high-quality information services, programs, food, and facilities enhance the hedonistic and practical aspects of the festival, thus elevating perceived value and satisfaction.

Emotions play a critical role in shaping tourists' perceptions, directly influencing service and experience evaluations (Sann et al., 2023). For example, when experiencing minor service issues, tourists in a positive emotional state are often less sensitive to these issues, maintaining higher satisfaction (Lee et al., 2014). Pine and Gilmore (1998) experience economy theory underscores that positive emotional experiences significantly boost overall satisfaction. Studies have also identified a positive relationship between emotions and satisfaction across different tourism settings (Leri and Theodoridis, 2019; Pestana et al., 2020).

Service management literature views satisfaction as an outcome of perceived value in a transaction or relationship (Seo and Um, 2023). From a social science perspective, cognitive processes can trigger emotional responses, implying that value judgments influence satisfaction perceptions (Yu et al., 2023). Tourism research often considers perceived value a precursor to satisfaction, viewing experience satisfaction as rooted in perceived value, encompassing both emotional and functional aspects (Konuk, 2019). For instance, in festival tourism, positive perceived value has been linked to heightened satisfaction and increased behavioral intentions (Perez-Monteagudo and Curras-Perez, 2022). Therefore, the following hypotheses are put forward:

H5. There is a positive relationship between existential authenticity and satisfaction.

H6. There is a positive relationship between experience quality and satisfaction.

H7. There is a positive relationship between tourist emotions and satisfaction.

H8. There is a positive relationship between perceived value and satisfaction.

### 2.7 Loyalty

Loyalty in the context of festival tourism refers to the likelihood of tourists returning to the event and recommending it to others (Girish and Chen, 2017). Tourist loyalty is considered fundamental for the stable growth of the festival tourism sector, including music festivals (Lee, 2016; Chi et al., 2022). Oliver (1999) conceptualizes loyalty as a multidimensional construct with stages: affective loyalty, cognitive loyalty, conative loyalty, and action loyalty. This study examines these loyalty dimensions within the context of music festivals.

Empirical research demonstrates that tourists' emotions can enhance loyalty (Choi and Kim, 2020; Godovykh and Tasci, 2021). For example, in a strawberry festival setting, tourists' emotions positively impacted loyalty by influencing satisfaction (Lee, 2014). Similarly, research on dance festival tourists indicates that both satisfaction and emotions positively correlate with loyalty (Lee et al., 2008).

Higher perceived value often leads to greater overall satisfaction, which is essential for building loyalty (Tanford, 2017; Lin et al., 2020). Strong evidence of the link between perceived value and loyalty exists across various tourism contexts, such as marine tourism (Su et al., 2021) and nature tourism (Zhou and Yu, 2022). Saha et al. (2023) found that at Norwegian music festivals, both functional and emotional values contribute to satisfaction and lead to behavioral intentions to revisit.

Evidence from multiple tourism domains underscores the significant influence of satisfaction on loyalty (Choi and Kandampully, 2019; Dai et al., 2023). Satisfied tourists are more likely to recommend the event to others and engage in positive word-of-mouth (Moon and Han, 2019). Wang et al. (2024) assert that in online music festival contexts, satisfaction with the experience is a critical precursor to loyalty. Therefore, this study proposes the following hypothesis:

H9. There is a positive relationship between tourist emotions and loyalty.

H10. There is a positive relationship between perceived value and loyalty.

H11. There is a positive relationship between satisfaction and loyalty.

**Figure 1:**
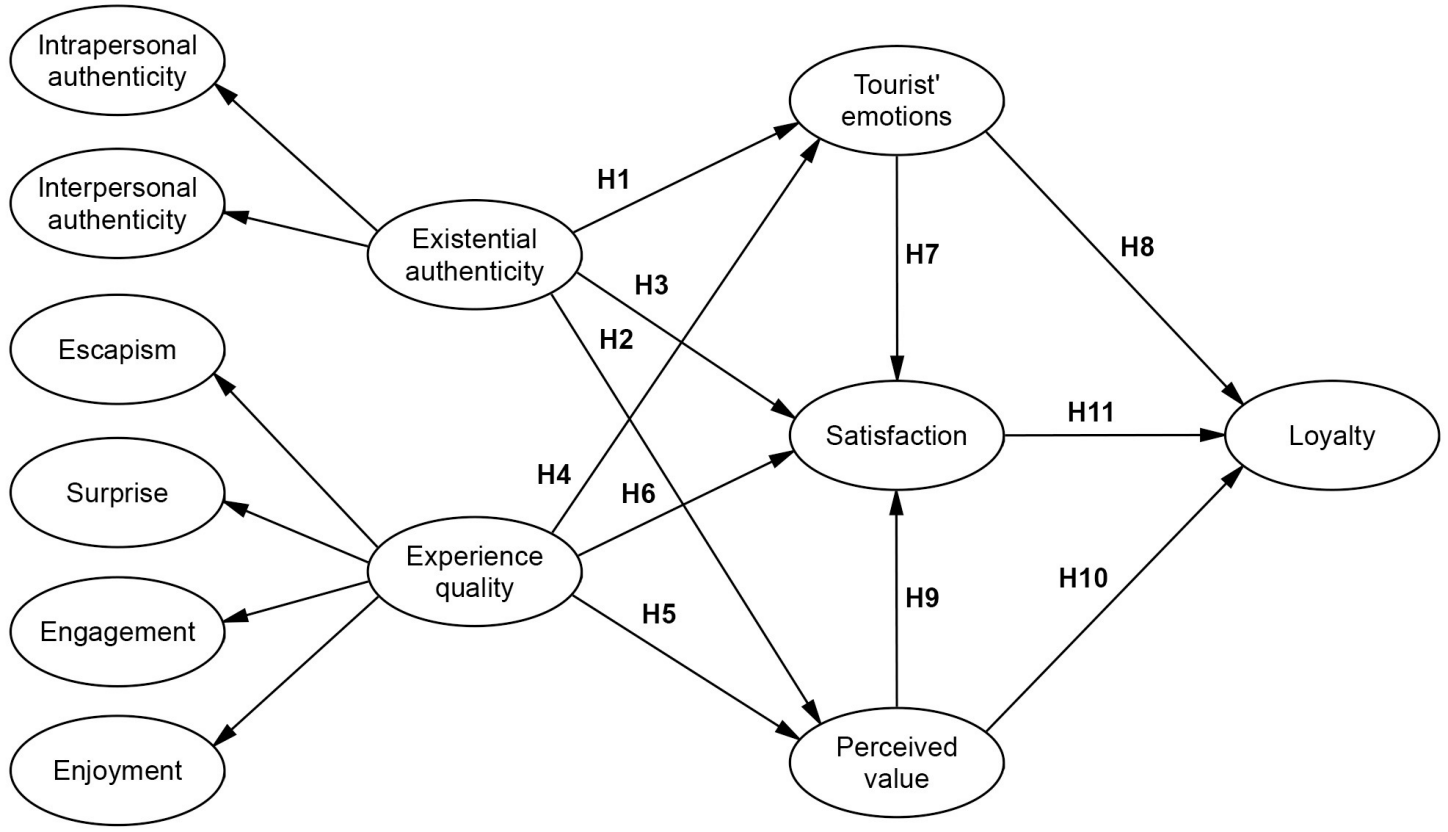
Conceptual model.

Grounded on the above research and assumptions, a conceptual model is presented in Figure 1.

## 3 Methodology

### 3.1 Research location and target respondents

The Strawberry Music Festival, a local event in China, has been hosted in major cities nationwide since early 2024. Its growth has not only increased brand recognition but also stimulated economic and cultural development in its host cities. The festival's themes of “spring, romance, and love” reflect attitudes and cultural ideals that resonate with many music enthusiasts, particularly the youth.

The research was conducted in Shenyang, Liaoning Province, a city known for its rich history, modern urban style, and thriving music scene (Liu et al., 2014). The 2023 Shenyang Strawberry Music Festival, branded as “Hello, Shenyang!” spanned two days and attracted over 40,000 attendees with its impressive lineup, striking stage design, and four vibrant performances. Approximately half of the visitors traveled from other cities, bringing a substantial boost to Shenyang's cultural, tourism, and economic sectors. This study focused on participants of the 2023 Shenyang Strawberry Music Festival. At the festival's end, researchers used convenience sampling at the exits to recruit departing attendees for data collection, a process that took 20 to 25 min per participant. Only individuals aged 18 and older were included in the study. Following Weston and Gore (2006) recommendation of a minimum of 200 samples for structural equation modeling, we collected 673 usable responses, exceeding the suggested sample size for robust data analysis.

### 3.2 Measurement and instrumentation design

Researchers designed a structured questionnaire by drawing on relevant prior studies, comprising seven sections: (1) existential authenticity; (2) experience quality; (3) tourist emotions; (4) perceived value; (5) festival satisfaction; (6) festival loyalty; and (7) basic demographic information. Existential authenticity was measured using eight items adapted from Hsu et al. (2021) and Zhao and Li (2023), divided evenly between interpersonal and intrinsic authenticity, and with adjustments made to fit the context of the Shenyang Strawberry Music Festival. The experience quality scale included 14 items validated by previous studies (Jin et al., 2015; Allan, 2016; Moon and Han, 2019). Tourist emotions were assessed using four items derived from Karagöz and Uysal (2022), while perceived value was measured with four items based on Yoon et al. (2010) and Jin et al. (2015). The satisfaction constructs also included four items adapted from prior festival studies (Yoon et al., 2010; Lee, 2016). Four items were integrated from research by Choi and Kandampully (2019) and Moon and Han (2019) to evaluate tourist loyalty. All items utilized a five-point Likert scale, ranging from “strongly disagree” (1) to “strongly agree” (5). In addition, the questionnaire included five single-choice and two multiple-choice questions to collect demographic data such as gender, age, education, income (in RMB), occupation, companions, and sources of information.

To ensure the applicability of these scales in the context of festival tourism in China, we adopted the translation and back-translation method for localization (Brislin, 1986). Additionally, three bilingual experts with backgrounds in tourism management and psychometrics were invited to proofread the language, in order to enhance semantic equivalence and cultural appropriateness. Before final data collection, a pilot test was conducted to confirm the reliability and validity of the questionnaire. The selection criteria for pilot test participants were consistent with those used in the main data collection. Visitors who had attended the 2023 Shenyang Strawberry Music Festival were asked if they would participate in this preliminary survey. Data for the pilot test were gathered on the first day of the festival using convenience sampling. A total of 91 respondents participated in the pre-survey, and the resulting data were used to assess the internal consistency and reliability of the variable scales. According to the criteria proposed by Nunnally and Bernstein (1994), Cronbach's alpha value >0.70 indicates that the scale has good internal consistency and reliability. The Cronbach's alpha values for the measured variables in this study ranged from 0.81 to 0.92, indicating good internal consistency and high scale reliability for all measured variables. Therefore, no items were deleted during the actual data collection process, and all 41 items were retained.

### 3.3 Data gathering and analysis

Data collection took place at the exits of the Shenyang Strawberry Music Festival, targeting attendees from August 26–27, 2023. Out of 756 festival-goers who participated in the survey, 673 responses were deemed usable, resulting in an effective response rate of ~89%. Based on the collected data, the sample had a slightly higher proportion of female respondents (60.77%) compared to male respondents (39.23%). Most participants (74.59%) had completed college or undergraduate education, with 9.06% holding or pursuing graduate degrees. Overall, 16.34% had not attained a college degree. In terms of income, 32.09% of respondents reported earnings below 5,001 RMB, while 67.91% had incomes above this threshold, with 23.33% earning more than 8,000 RMB. The average age of participants ranged between 18 and 40, reflecting a younger demographic. This aligns with previous research on music festivals, where over 90% of attendees were found to be less than 40 years old. The participants represented various occupations, with students (20.06%) and freelancers (25.11%) making up a significant portion. Most attendees came to the festival with their spouses (39.23%) or friends (47.55%). Information about the festival was obtained through diverse channels, predominantly video platforms like Douban, Kugou, and Bilibili (62.41%), as well as online communities such as Xiaohongshu and Weibo (57.95%). Table 1 provides further details on the demographic characteristics of the respondents.

**Table 1:** Research participants' general characteristics.

**Variable**	**Category**	**Number**	**%**
Gender	Female	409	60.77
	Male	264	39.23
Age (years)	18–30	525	78.01
	31–40	131	19.47
	41–60	17	2.53
	Over 60	0	0
Education	Junior high school or below	11	1.63
	High school or technical secondary school	99	14.71
	College or undergraduate	502	74.59
	Graduate student	61	9.06
Income (CNY)	Less than 2,000	84	12.48
	2,001–5,000	132	19.61
	5,001–8,000	300	44.58
	8,001–15,000	126	18.72
	Over 15,000	31	4.61
Profession	Enterprise unit worker	129	19.17
	Civil servants, public institution workers	97	14.41
	Self-employed	113	16.79
	Freelancer	169	25.11
	Farmer or worker	23	3.42
	Student	135	20.06
	Retiree	1	0.15
	Else	6	0.89
Companions	Couple or spouse	264	39.23
	Children	41	6.09
	Parent	55	8.17
	Friends	320	47.55
	Colleagues	109	16.2
	Lone wolf	58	8.62
	Else	12	1.78
Information receiving channels	Small red book, Weibo, and other online communities	390	57.95
	Recommended by friends and family	258	38.34
	Advertisements, newspapers, magazines	208	30.91
	Video platforms such as Douyin, Kuaishou, and B station	420	62.41
	WeChat, QQ, and other social software	294	43.68
	Travel agencies and other tourism organizations	120	17.83
	Baidu, Google, and other search engines	77	11.44
	Ctrip and other travel websites	45	6.69
	Else	5	0.74

1 CNY equals ~0.14 USD.

Data analysis was conducted using SPSS 27.0 and AMOS 26.0. Initially, a Confirmatory Factor Analysis (CFA) was performed to assess the reliability and validity of the measurement instruments. Cronbach's alpha coefficients were also analyzed to verify the internal consistency of each subscale, thus evaluating the measurement model. Structural Equation Modeling (SEM) was then employed to test the research model. Finally, the analysis included the calculation of direct, indirect, and total effects among the variables. The efficacy and strength of our SEM were meticulously evaluated using a range of well-established fit indices, including the Chi-Square to Degrees of Freedom Ratio, the Tucker-Lewis Index (TLI), the Comparative Fit Index (CFI), the Standardized Root Mean Square Residual (SRMR), and the Root Mean Square Error (RMSE) of Approximation (Schermelleh-Engel et al., 2003). A model was considered adequate when the TLI and the CFI reached or exceeded 0.95, and SRMR as well as the RMSE of Approximation remained below 0.05, thereby affirming the model's validity and reliability (Schermelleh-Engel et al., 2003).

## 4 Results

### 4.1 Measurement modeling

According to the standards outlined by Whittaker and Schumacker (2022), standardized factor loadings should not fall below 0.50. While all items in this study met this criterion, ESC4, EBJ4, and SAT1 were removed to improve model fit, despite their standardized loadings being above 0.70 (ranging from 0.72 to 0.90). The decision to remove these items was based on multiple model diagnostics, including modification indices (MIs > 15) and item redundancy observed during confirmatory factor analysis (CFA). Specifically, these items contributed to cross-loadings or localized strain within their constructs, and their removal led to substantial improvements in key fit indices without compromising the conceptual integrity of the latent constructs. This approach is consistent with the recommendation by Hair et al. (2019) that theoretically redundant or statistically problematic items may be removed for better parsimony and model performance, as long as the core meaning of the constructs is preserved.

Following Segars (1997), the composite reliability (CR) for all variables should exceed 0.70, and the average variance extracted (AVE) should be >0.50. All 10 variables in this study satisfied these requirements (see Table 2).

**Table 2:** Analysis results of construct validity and reliability.

**Constructs/ Variables**		**Mean**	**Std. dev**	**Factor loadings**	**α**	**CR**	**AVE**
Intrapersonal authenticity		3.07	0.97		0.896	0.879	0.658
INTRA1	I am able to discover more about myself.			0.822			
INTRA2	I am in touch with my feelings and emotions.			0.809			
INTRA3	I was freed from the limitations of daily work or routine life and became more self and subjective in my own right.			0.830			
INTRA4	I tried to seek extraordinary experiences to pursue self-realization or get self-satisfaction.			0.848			
Interpersonal authenticity		3.05	0.94		0.890	0.890	0.669
INTER1	I have contact with performers in a natural, authentic, and friendly way.			0.821			
INTER2	I have contact with family members in a natural, authentic, and friendly way.			0.795			
INTER3	I have contact with other tourists in a natural, authentic, and friendly way.			0.821			
INTER4	I have contact with workers in a natural, authentic, and friendly way.			0.834			
Escapism		3.64	1.04		0.799	0.800	0.572
ESC1	I felt I played a different character here.			0.773			
ESC2	I felt like I was living in a different time or place.			0.731			
ESC3	The experience here let me imagine being someone else.			0.764			
Surprise		3.72	0.98		0.846	0.847	0.580
SUR1	The events at this music festival are special.			0.764			
SUR2	The service at this music festival is consistent and reliable.			0.749			
SUR3	There are some unexpected and unique performances.			0.720			
SUR4	The service at this music festival makes me feel special and valued.			0.811			
Participation		3.67	1.00		0.855	0.856	0.598
PAR1	I appreciated all the performances or as many as possible.			0.803			
PAR2	I participated in activities that the music festival provided.			0.756			
PAR3	I want to be a member of this music festival to receive benefits.			0.741			
PAR4	I can choose any activities which are suitable for me.			0.792			
Enjoyment		3.70	0.99		0.796	0.797	0.567
ENJ1	I felt that I was doing something I really liked to do at this music festival.			0.776			
ENJ2	I felt that I was doing something memorable at this music festival.			0.729			
ENJ3	I felt that I was having fun at this music festival.			0.753			
Tourist emotions		3.26	0.98		0.857	0.857	0.600
TE1	I feel pleasant.			0.738			
TE2	I feel arousing.			0.765			
TE3	I feel excited.			0.732			
TE4	I feel relaxed.			0.857			
Perceived value		3.26	0.99		0.865	0.863	0.613
PV1	This music festival was worth the money, time, and effort I spent.			0.744			
PV2	This music festival offered more value than expected.			0.768			
PV3	This music festival offered more value than other festivals.			0.747			
PV4	This music festival's overall quality of service was valuable.			0.865			
Satisfaction		3.33	0.98		0.914	0.912	0.723
SAT2	This music festival offers me more value than I expected.			0.816			
SAT3	I believe I did the right thing in attending this music festival.			0.881			
SAT4	My choice to visit this music festival was a wise one.			0.897			
SAT5	As a whole, I am happy with this music festival.			0.803			
Loyalty		3.36	0.92		0.884	0.885	0.659
LOY1	I will say positive things about the music festival to other people.			0.784			
LOY2	I will keep attending the music festival if it is held again in the future.			0.741			
LOY3	I will recommend the music festival to my relatives and friends.			0.836			
LOY4	I will take pride in telling other people about my experiences at the music festival.			0.878			

INTRA, intrapersonal authenticity; INTER, interpersonal authenticity; ESC, escapism; SUR, surprise; PAR, participation; ENJ, enjoyment; TE, tourist emotions; PV, perceived value; SAT, satisfaction; LOY, loyalty.

Regarding discriminant validity, the square root of each variable's AVE must be higher than the correlation coefficients between that variable and others (Fornell and Larcker, 1981). The measurement model in this study met this standard, indicating strong discriminant validity (see Table 3). Based on the criteria recommended in Table 4, the results showed that the model fit was satisfactory, with χ^2^*/df* (variance/degrees of freedom) = 2.351, AGFI (adjusted goodness-of-fit index) = 0.865, TLI (Tucker-Lewis index) = 0.931, CFI (comparative fit index) = 0.937, IFI (incremental fit index) = 0.937, and RMSEA (root mean square error of approximation) = 0.045 (see Table 4).

**Table 3:** Discriminate validity of the research model.

**Constructs**	**INTRA**	**INTER**	**ESC**	**SUR**	**PAR**	**ENJ**	**TE**	**PV**	**SAT**	**LOY**
INTRA	0.811									
INTER	0.548	0.818								
ESC	0.147	0.115	0.756							
SUR	0.133	0.101	0.388	0.762						
PAR	0.148	0.093	0.433	0.409	0.773					
ENJ	0.112	0.130	0.403	0.403	0.379	0.753				
TE	0.166	0.206	0.202	0.204	0.202	0.266	0.775			
PV	0.243	0.236	0.330	0.262	0.247	0.247	0.233	0.783		
SAT	0.217	0.260	0.307	0.295	0.219	0.262	0.297	0.506	0.850	
LOY	0.080	0.134	0.079	0.087	0.106	0.070	0.142	0.294	0.375	0.812

INTRA, intrapersonal authenticity; INTER, interpersonal authenticity; ESC, escapism; SUR, surprise; PAR, participation; ENJ, enjoyment; TE, tourist emotions; PV, perceived value; SAT, satisfaction; LOY, loyalty.

**Table 4:** The goodness of fit indices for the measurement model and research model.

**Model**	***x* ^2^**	***x^2^/df***	**AGFI**	**TLI**	**CFI**	**IFI**	**RMSEA**
Measurement model	1,509.615 (0.000)	2.351	0.865	0.931	0.937	0.937	0.045
Research model	754.384 (0.000)	1.164	0.937	0.992	0.992	0.992	0.016
Recommended criteria	*P* > 0.05	<5.0	>0.90	>0.90	>0.90	>0.90	<0.08

### 4.2 Structural modeling

The research model was validated using Structural Equation Modeling (SEM). The model fit indices showed excellent fit, with χ^2^*/df* = 1.164, AGFI = 0.937, TLI = 0.992, CFI = 0.992, IFI = 0.992, and RMSEA = 0.016 (see Table 4). The hypothesis testing results indicated that 10 out of 11 hypotheses were supported, except for H8 (see Table 5).

**Table 5:** The results hypotheses test.

**Hypotheses**	**Hypothesized path**	***B***	**β**	**S.E**	***t***	**Result**
H1	EA → TE	0.250	0.203	0.059	4.208^***^	Supported
H2	EA → PV	0.383	0.269	0.068	5.630^***^	Supported
H3	EA → SAT	0.185	0.142	0.058	3.183^**^	Supported
H4	EQ → TE	0.430	0.350	0.064	6.744^***^	Supported
H5	EQ → PV	0.611	0.430	0.074	8.253^***^	Supported
H6	EQ → SAT	0.239	0.184	0.067	3.554^***^	Supported
H7	TE → SAT	0.127	0.120	0.044	2.861^**^	Supported
H8	TE → LOY	0.012	0.013	0.042	0.296	Rejected
H9	PV → SAT	0.367	0.401	0.043	8.476^***^	Supported
H10	PV → LOY	0.123	0.144	0.043	2.865^**^	Supported
H11	SAT → LOY	0.296	0.318	0.048	6.126^***^	Supported

EA, existential authenticity; EQ, experience quality; TE, tourist emotions; PV, perceived value; SAT, satisfaction; LOY, loyalty. ^***^p <0.001, ^**^p <0.01.

**Figure 2:**
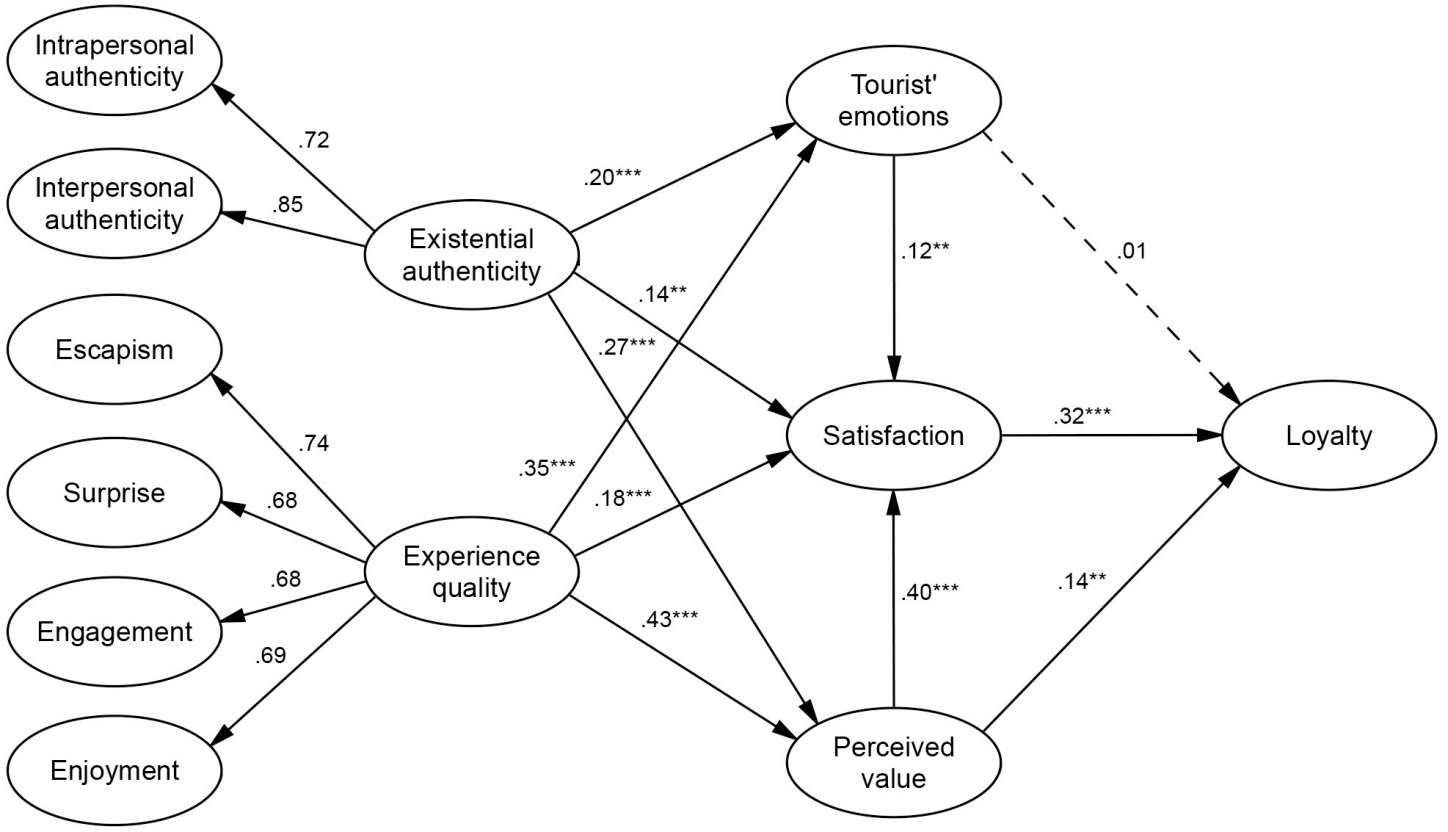
Structural model. ***Significant at *p* < 0.001, **Significant at *p* < 0.01.

Figure 1 illustrates the structural model of this study. Specifically, “existential authenticity” had a significant positive correlation with “tourist emotions” (β = 0.203, *t* = 4.208, *p* < 0.001), “satisfaction” (β = 0.142, *t* = 3.183, *p* < 0.01), and “perceived value” (β = 0.269, *t* = 5.630, *p* < 0.001). “Experience quality” also showed a significant positive correlation with “tourist emotions” (β = 0.350, *t* = 6.744, *p* < 0.001), “satisfaction” (β = 0.184, *t* = 3.554, *p* < 0.001), and “perceived value” (β = 0.430, *t* = 8.253, *p* < 0.001). Additionally, “tourist emotions” were positively associated with “satisfaction” (β = 0.120, *t* = 2.861, *p* < 0.001), and “perceived value” had a significant positive relationship with “satisfaction” (β = 0.401, *t* = 8.476, *p* < 0.001). The validated structural model is shown in Figure 2.

### 4.3 Direct, indirect, and aggregate effects between variables

After removing the non-significant paths, the direct, indirect, and total effects between variables such as existential authenticity, experience quality, tourist emotions, perceived value, satisfaction, and loyalty were recalculated (see Table 6). The results showed that the indirect effect of existential authenticity on loyalty, mediated by tourist emotions, perceived value, and satisfaction, had a weight of 0.129. Similarly, the indirect effect of experience quality on loyalty through the same mediators was 0.193.

**Table 6:** Direct, indirect, and total effects among the variables.

**Dependent variable**	**Independent variable**	**Direct effect**	**Indirect effect**	**Total effect**	***R^2^***
INTRA	EA	0.717^***^	–	0.717	0.514
INTER	EA	0.854^***^	–	0.854	0.730
ESC	EQ	0.738^***^	–	0.738	0.544
SUR	EQ	0.682^***^	–	0.682	0.465
PAR	EQ	0.678^***^	–	0.678	0.459
ENJ	EQ	0.694^***^	–	0.694	0.481
TE	EA	0.203^***^	–	0.203	0.164
	EQ	0.350^***^	–	0.350	
PV	EA	0.269^***^	–	0.269	0.257
	EQ	0.430^***^	–	0.430	
SAT	EA	0.142^**^	0.132	0.275	0.366
	EQ	0.184^***^	0.214	0.398	
	TE	0.120^**^	–	0.120	
	PV	0.401^***^	–	0.401	
LOY	EA	–	0.129	0.129	0.175
	EQ	–	0.193	0.193	
	TE	0.013	0.038	0.051	
	PV	0.144^**^	0.128	0.272	
	SAT	0.318^***^	–	0.318	

EA, existential authenticity; EQ, experience quality; INTRA, intrapersonal authenticity; INTER, interpersonal authenticity; ESC, escapism; SUR, surprise; PAR, participation; ENJ, enjoyment; TE, tourist emotions; PV, perceived value; SAT, satisfaction; LOY, loyalty. ^***^p <0.001, ^**^p <0.01.

Most of the indirect effects were primarily mediated by perceived value (β = 0.430 × 0.401 = 0.173), while only a small portion was mediated by tourist emotions (β = 0.203 × 0.013 = 0.003).

## 5 Discussion and conclusions

### 5.1 Research conclusions

This study is the first to use the S-O-R paradigm to explore factors influencing festivalgoer loyalty, focusing on the roles of existential authenticity, experience quality, tourist emotions, perceived value, and satisfaction. In the post-pandemic context, where domestic tourism and large-scale events are recovering, understanding how these factors shape loyalty is crucial.

First, the study found that existential authenticity and experience quality, as key stimuli, have significant positive effects on tourists' emotions, perceived value, and satisfaction. This indicates that when festivalgoers experience a stronger sense of authenticity and high-quality events, they tend to have more positive emotions, enhancing their overall perceived value and satisfaction with the festival. Especially in the post-pandemic era, there is a heightened desire to escape from daily routines and seek authentic, unique experiences, amplifying the impact of existential authenticity on tourists' emotions and behaviors (Cai et al., 2022; Yao et al., 2023). Notably, existential authenticity not only affects emotional experiences but also indirectly enhances loyalty through perceived value. This result aligns with previous studies in festival tourism, supporting the broad influence of existential authenticity on tourist decision-making (Hsu et al., 2021; Fang and Liu, 2024). Furthermore, cultural factors may further influence the relationships among variables. Chinese culture emphasizes interpersonal interactions, social connections, and a sense of group belonging (Zhao and Li, 2023); therefore, the impact of interpersonal authenticity on tourist satisfaction and loyalty may be more pronounced. In contrast, in Western culture, individuals place greater emphasis on self-expression and unique experiences (Matheson, 2008), and the influence of existential authenticity on loyalty may rely more on intrapersonal authenticity. This cultural difference warrants further cross-cultural comparisons in future research to explore in depth the moderating role of culture on festival tourism experiences and tourist behavior.

Second, experience quality was identified as a powerful stimulus. The study confirms that high-quality music festival experiences significantly boost tourists' emotions and perceived value, consistent with prior research on the positive impacts of tourism experience quality on emotions and behavior (Yoon et al., 2010; Wu et al., 2018; Özdemir et al., 2023). Post-pandemic Chinese music festivals blend rich local culture with modern urban elements, creating unique experiences for attendees. For instance, Shenyang, a city with a rich historical background and modern appeal, adds a distinctive cultural blend to the festival (Liu et al., 2014). Visitors not only enjoy diverse musical performances but also engage in various interactive activities, creating “moments of surprise” that effectively elicit positive emotions. This cultural fusion may explain why festivals can evoke high emotional responses and satisfaction. Additionally, perceived value serves as an essential mediator between experience quality and loyalty, suggesting that high-quality experiences not only enhance perceived value but also strengthen festivalgoer loyalty.

However, the study did not find a significant direct effect of tourist emotions on loyalty, which contradicts several previous studies (Choi and Kim, 2020; Godovykh and Tasci, 2021). This unexpected result merits further exploration. One possible explanation lies in the temporal misalignment between short-term emotions and long-term loyalty. Emotions experienced during a festival are typically transient, situational, and highly sensitive to environmental factors such as weather, crowd density, or performance quality (Lee et al., 2011). Loyalty, by contrast, represents a stable attitudinal and behavioral commitment formed over time, which may not be immediately affected by fleeting emotional states (Moon and Han, 2019).

Additionally, it is possible that festivalgoers process emotional experiences retrospectively—that is, their emotional evaluation is only fully formed after the event concludes (Godovykh and Tasci, 2021). As such, their responses in post-event surveys may prioritize more cognitive assessments like satisfaction and perceived value, rather than recalling specific emotional highs or lows. Another interpretation is that emotional arousal alone is insufficient to drive loyalty unless it is accompanied by cognitive appraisal and evaluative judgment—such as the perception that the experience was valuable or exceeded expectations (Ampadu et al., 2023). This is consistent with dual-process theories in psychology, which suggest that affective responses require cognitive reinforcement to influence long-term behavioral intentions (Jang et al., 2021).

Finally, this study enriches the understanding of Chinese music tourism in the post-pandemic era and offers practical insights for festival organizers and destination managers. By enhancing existential authenticity and experience quality, they can effectively boost perceived value, leading to higher satisfaction and loyalty. This approach is essential for the recovery and sustainable development of music festivals in China in the post-pandemic era.

### 5.2 Theoretical contributions

While the study is context-specific, it offers theoretical contributions that may inspire broader applications in festival tourism research. This study extends the application of the Stimulus-Organism-Response (S-O-R) theory in the field of festival tourism. Previous research on music festivals has predominantly relied on the experience economy theory (Li and Wood, 2016; Tan et al., 2023); in contrast, this study introduces existential authenticity as an environmental stimulus to elucidate its influence on tourist emotions, perceived value, satisfaction, and loyalty. The results indicate that existential authenticity, together with experience quality, affects tourist emotions, and perceived value not only directly but also indirectly influences satisfaction and loyalty through perceived value, thereby enriching the conceptualization of the “stimulus” component within the S-O-R framework.

Furthermore, this study refines the theoretical role of existential authenticity in the context of music festivals. Previous studies (e.g., Su et al., 2021; Fu, 2019) have largely focused on authenticity experiences in heritage tourism or natural attractions; however, this study is the first to demonstrate its significant role in the highly emotive and experiential setting of music festivals—especially in the post-pandemic era when tourists' heightened needs for self-expression and identity recognition render the impact of existential authenticity on emotions and satisfaction even more pronounced. This broadens the scope of authenticity theory and offers new perspectives for future research.

Finally, this study confirms the indirect role of emotions in the formation of loyalty in festival tourism, thereby challenging or revising previous assumptions. While previous research (e.g., Godovykh and Tasci, 2021; Lee et al., 2014) suggested that positive emotions directly enhance loyalty, our findings reveal that emotions do not exert a direct effect; rather, they influence loyalty indirectly through perceived value and satisfaction. This suggests that tourist emotions at music festivals are more transient and volatile, with their impact on loyalty relying more on cognitive evaluations (i.e., perceived value) and overall assessments (i.e., satisfaction). This finding broadens the applicability of emotion theory in the realm of tourism experiences and underscores the need for future research to examine the interplay between emotional and cognitive factors.

### 5.3 Management implications

From a managerial perspective, this study provides practical guidance for the sustainable development of music festivals and destination marketing, particularly in the context of post-pandemic recovery. Firstly, music festival organizers should enhance visitors' experience of existential authenticity. This can be achieved through immersive interactive projects such as art installations, immersive performances, and creative interactive activities that help tourists explore and express themselves. Additionally, creating a free and open social environment—for example, dedicated social zones, themed parties, and interactive games—will foster genuine communication and enhance overall satisfaction. Secondly, the quality of the overall experience should be improved. This includes optimizing service facilities to ensure high quality and reliability, as well as planning creative activities such as limited-edition souvenirs, guest interactions, and special artistic performances. Moreover, offering personalized services, such as VIP experiences, reservation services, or interest-based community interactions, can further enhance visitor engagement and satisfaction. Thirdly, attention should be paid to visitors' emotional experiences. By employing data analysis and social media interactions to accurately capture tourist preferences, organizers can tailor personalized music programs and interactive experiences (such as virtual reality experiences). At the same time, optimizing environmental design by creating leisure areas, social interaction zones, or spaces for emotional relief will boost visitor satisfaction and their willingness to return. Finally, local cultural and tourism management departments can promote the integration of music festivals with local culture, historical attractions, and traditional crafts to create culturally distinctive, environmentally friendly, and community-oriented festival events. This strategy not only enhances the appeal of music festivals but also extends visitor dwell time, fosters local economic development, and contributes to the long-term benefits and reputation of the tourism industry.

To evaluate the practical effectiveness of these strategies, organizers can implement systematic feedback mechanisms, such as regular surveys on visitor satisfaction and loyalty across different experience dimensions. Additionally, organizers can track and analyze repeat visitation rates and willingness to recommend the festival, thereby quantifying loyal behavior. Continuously monitoring visitor feedback on social media and online platforms and optimizing management measures based on real-time evaluations is also an effective approach. These methods will help organizers enhance the festival experience quality and visitor loyalty, thereby promoting the sustainable development of music festival events.

## 6 Limitations and future research

Although this study makes contributions to both theory and practice, there are still several limitations. First, data collection was based solely on a field survey of the Shenyang Strawberry Music Festival, and the sample region is relatively homogeneous, which may limit the external validity and generalizability of the research findings. Music festivals in different regions and cultural contexts may exhibit significant differences in terms of tourist interactions, emotional expressions, and experience preferences. This is especially true in international contexts, where tourists' perceptions of existential authenticity and its impact on emotions and satisfaction may differ (Tan et al., 2020). Therefore, future research could consider cross-regional and cross-cultural comparative studies to enhance the generalizability and applicability of the research conclusions.

Secondly, in terms of variable selection, this study did not include key demographic variables (such as age, gender, prior participation experience, and music preferences), nor did it account for external environmental factors (such as social media dissemination, marketing strategies, and the macroeconomic context). This omission may, to some extent, limit a comprehensive explanation of variations in tourist behavior. Previous studies have indicated that different demographic characteristics and contextual factors can significantly influence tourists' perceptions of authenticity, emotional responses, and behavioral intentions (Chi, 2010; Leenders, 2010; Raab et al., 2016). Future research should incorporate a broader range of variables into theoretical modeling to construct a more comprehensive analytical framework that integrates both individual psychological factors and external environmental influences.

Third, this study employed a convenience sampling method, conducting surveys at the exit of the festival at the end of the event, which may introduce sample bias. For instance, it is more likely that we engaged with visitors who had a positive experience and were willing to stay until the event concluded, while those who left early or had a less favorable experience may have been overlooked. At the same time, self-selection bias should not be ignored—satisfied visitors are more likely to participate in the survey, whereas dissatisfied ones may choose not to respond (Smironva et al., 2020). Future research could adopt a multi-phase, multi-channel sampling strategy that integrates both online and offline methods, and incorporate random sampling or big data analysis (such as social media comment mining) to enhance the representativeness of the sample and the robustness of the findings.

Finally, the mechanisms underlying the formation of visitor loyalty may vary depending on visitor type. First-time visitors are often more influenced by external stimuli, novelty, or marketing efforts, whereas the loyalty of repeat visitors tends to stem from accumulated past experiences, emotional identification, and value resonance (Jin et al., 2015). Future studies could incorporate this distinction into sample design to explore in depth the similarities and differences in loyalty drivers across different types of visitors.

## Data Availability

The raw data supporting the conclusions of this article will be made available by the authors, without undue reservation.

## References

[B1] Ahn J. Kwon J. (2020). Green hotel brands in Malaysia: perceived value, cost, anticipated emotion, and revisit intention. Curr. Issues Tour. 23, 1559–1574. 10.1080/13683500.2019.1646715

[B2] Allan M. (2016). Place attachment and tourist experience in the context of desert tourism – the case of wadi rum. Czech J. Tour. 5, 35–52. 10.1515/cjot-2016-0003

[B3] Ampadu S. Jiang Y. Gyamfi S. A. Debrah E. Amankwa E. (2023). Perceived value of recommended product and consumer e-loyalty: an expectation confirmation perspective. Young Consum. 24, 742–766. 10.1108/YC-08-2022-1597

[B4] Armbrecht J. (2021). Event quality, perceived value, satisfaction and behavioural intentions in an event context. Scand. J. Hosp. Tour. 21, 169–191. 10.1080/15022250.2021.1877191

[B5] Aşan K. Kaptangil K. Gargaci Kinay A. (2020). Mediating role of perceived festival value in the relationship between experiences and satisfaction. Int. J. Event Festiv. Manag. 11, 255–271. 10.1108/IJEFM-11-2019-0058

[B6] Atzeni M. Del Chiappa G. Mei Pung J. (2022). Enhancing visit intention in heritage tourism: the role of object-based and existential authenticity in non-immersive virtual reality heritage experiences. Int. J. Tour. Res. 24, 240–255. 10.1002/jtr.2497

[B7] Bagozzi R. P. Gopinath M. Nyer P. U. (1999). The role of emotions in marketing. J. Acad. Mark. Sci. 27, 184–206. 10.1177/0092070399272005

[B8] Brislin R. W. (1986). “The wording and translation of research instruments,” in Field Methods in Cross-Cultural Research, eds. W. Lonner and J. Berry (Beverly Hills, CA: Sage), 37–164.

[B9] Brown A. E. Sharpley R. (2019). Understanding festival-goers and their experience at UK music festivals. Event Manag. 23, 699–720. 10.3727/152599519X15506259855733

[B10] Cai Y. Li G. Liu C. Wen L. (2022). Post-pandemic dark tourism in former epicenters. Tour. Econ. 28, 175–199. 10.1177/13548166211034639

[B11] Chaney D. Martin D. (2017). The role of shared values in understanding loyalty over time: a longitudinal study on music festivals. J. Travel Res. 56, 507–520. 10.1177/0047287516643411

[B12] Chang J.-J. Chen R.-F. Lin C.-L. (2022). Exploring the driving factors of urban music festival tourism and service development strategies using the modified SIA-NRM approach. Sustainability 14:7498. 10.3390/su14127498

[B13] Chen C.-F. Chen Y.-X. (2023). Investigating the effects of platform and mobility on mobility as a service (MaaS) users' service experience and behavioral intention: empirical evidence from MeNGo, Kaohsiung. Transportation 50, 2299–2318. 10.1007/s11116-022-10309-5

[B14] Chen G. So K. K. F. Hu X. Poomchaisuwan M. (2022). Travel for affection: a stimulus-organism-response model of honeymoon tourism experiences. J. Hosp. Tour. Res. 46, 1187–1219. 10.1177/10963480211011720

[B15] Chen Q. Huang R. Hou B. (2020). Perceived authenticity of traditional branded restaurants (China): impacts on perceived quality, perceived value, and behavioural intentions. Curr. Issues Tour. 23, 2950–2971. 10.1080/13683500.2020.1776687

[B16] Chi C. G. (2010). Destination loyalty formation and travelers' demographic characteristics: a multiple group analysis approach. J. Hosp. Tour. Res. 35, 191–212. 10.1177/1096348010382233

[B17] Chi X. Han H. (2021). Emerging rural tourism in China's current tourism industry and tourist behaviors: the case of Anji County. J. Travel Tour. Mark. 38, 58–74. 10.1080/10548408.2020.1862026

[B18] Chi X. Meng B. Zhou H. Han H. (2022). Cultivating and disseminating a festival image: the case of the Qingdao International Beer Festival. J. Travel Tour. Mark. 39, 373–393. 10.1080/10548408.2022.2105474

[B19] Choi B. Kim H. S. (2020). Customer-to-customer interaction quality, promotion emotion, prevention emotion and attitudinal loyalty in mass services. J. Serv. Theory Pract. 30, 257–276. 10.1108/JSTP-08-2019-0172

[B20] Choi H. Kandampully J. (2019). The effect of atmosphere on customer engagement in upscale hotels: an application of S-O-R paradigm. Int. J. Hosp. Manag. 77, 40–50. 10.1016/j.ijhm.2018.06.012

[B21] Dai Q. Peng S. Guo Z. Zhang C. Dai Y. Hao W.. (2023). Place identity as a mediator between motivation and tourist loyalty in ‘red tourism.' *PLoS ONE* 18:e0284574. 10.1371/journal.pone.0284574 37889893 PMC10610084

[B22] Domínguez-Quintero A. M. González-Rodríguez M. R. Roldán J. L. (2019). The role of authenticity, experience quality, emotions, and satisfaction in a cultural heritage destination. J. Herit. Tour. 14, 491–505. 10.1080/1743873X.2018.1554666

[B23] Fakfare P. Lee J.-S. Kim J. J. Ryu H. B. Han H. (2024). Animal ethics and tourism: deepening a stimulus–organism–response (S-O-R) framework. J. Travel Res. 63, 940–958. 10.1177/00472875231175079

[B24] Fang Y.-P. Liu C.-H. (2024). Unraveling the difference mechanism of authenticity experience in determining antecedents and consequences of destination loyalty. J. Travel Tour. Mark. 41, 849–863. 10.1080/10548408.2024.2350694

[B25] Fornell C. Larcker D. F. (1981). Structural Equation Models With Unobservable Variables and Measurement Error: Algebra And Statistics. Ann Arbor, MI: Graduate School of Business Administration, The University of Michigan.

[B26] Fu X. (2019). Existential authenticity and destination loyalty: evidence from heritage tourists. J. Destin. Mark. Manag. 12, 84–94. 10.1016/j.jdmm.2019.03.008

[B27] Genc V. Gulertekin Genc S. (2023). The effect of perceived authenticity in cultural heritage sites on tourist satisfaction: the moderating role of aesthetic experience. J. Hosp. Tour. Insights 6, 530–548. 10.1108/JHTI-08-2021-0218

[B28] Girish V. G. Chen C.-F. (2017). Authenticity, experience, and loyalty in the festival context: evidence from the San Fermin festival, Spain. Curr. Issues Tour. 20, 1551–1556. 10.1080/13683500.2017.1296821

[B29] Godovykh M. Tasci A. D. A. (2021). The influence of post-visit emotions on destination loyalty. Tour. Rev. 76, 277–288. 10.1108/TR-01-2020-0025

[B30] Hair J. F. Risher J. J. Sarstedt M. Ringle C. M. (2019). When to use and how to report the results of PLS-SEM. Eur. Bus. Rev. 31, 2–24. 10.1108/EBR-11-2018-0203

[B31] Ho J. M. Tiew F. Adamu A. A. (2022). The determinants of festival participants' event loyalty: a focus on millennial participants. Int. J. Event Festiv. Manag. 13, 422–439. 10.1108/IJEFM-01-2022-0006

[B32] Hsu F.-C. Agyeiwaah E. Chen L. I.-L. (2021). Examining food festival attendees' existential authenticity and experiential value on affective factors and loyalty: an application of stimulus-organism-response paradigm. J. Hosp. Tour. Manag. 48, 264–274. 10.1016/j.jhtm.2021.06.014

[B33] Jang W. Kim J. Kim S. Chun J. W. (2021). The role of engagement in travel influencer marketing: the perspectives of dual process theory and the source credibility model. Curr. Issues Tour. 24, 2416–2420. 10.1080/13683500.2020.1845126

[B34] Jin N. P. Lee S. Lee H. (2015). The effect of experience quality on perceived value, satisfaction, image and behavioral intention of water park patrons: new versus repeat visitors: the effect of experience quality. Int. J. Tour. Res. 17, 82–95. 10.1002/jtr.1968

[B35] Karagöz D. Uysal M. (2022). Tourists' need for uniqueness as a representation of differentiated identity. J. Travel Res. 61, 76–92. 10.1177/0047287520972804

[B36] Kato T. (2021). Functional value vs emotional value: a comparative study of the values that contribute to a preference for a corporate brand. Int. J. Inf. Manag. Data Insights 1:100024. 10.1016/j.jjimei.2021.100024

[B37] Kim J.-H. (2022). Destination attributes affecting negative memory: scale development and validation. J. Travel Res. 61, 331–345. 10.1177/0047287520977725

[B38] Kim M. J. Lee C.-K. Jung T. (2020). Exploring consumer behavior in virtual reality tourism using an extended stimulus-organism-response model. J. Travel Res. 59, 69–89. 10.1177/0047287518818915

[B39] Konuk F. A. (2019). The influence of perceived food quality, price fairness, perceived value and satisfaction on customers' revisit and word-of-mouth intentions towards organic food restaurants. J. Retail. Consum. Serv. 50, 103–110. 10.1016/j.jretconser.2019.05.005

[B40] Kruger M. Saayman M. (2019). ‘All that jazz': the relationship between music festival visitors' motives and behavioural intentions. Curr. Issues Tour. 22, 2399–2414. 10.1080/13683500.2018.1451496

[B41] Laros F. J. M. Steenkamp J.-B. E. M. (2005). Emotions in consumer behavior: a hierarchical approach. J. Bus. Res. 58, 1437–1445. 10.1016/j.jbusres.2003.09.01339905339

[B42] Lee J. (2014). Visitors' emotional responses to the festival environment. J. Travel Tour. Mark. 31, 114–131. 10.1080/10548408.2014.861726

[B43] Lee J. S. Lee C. K. Choi Y. (2011). Examining the role of emotional and functional values in festival evaluation. J. Travel Res. 50, 685–696. 10.1177/0047287510385465

[B44] Lee S. Kim M. Kim H. (2024). Relationality of objective and constructive authenticities: effects on existential authenticity, memorability, and satisfaction. J. Travel Res. 63, 195–214. 10.1177/00472875221143468

[B45] Lee S. Phau I. Hughes M. Li Y. F. Quintal V. (2016). Heritage tourism in Singapore Chinatown: a perceived value approach to authenticity and satisfaction. J. Travel Tour. Mark. 33, 981–998. 10.1080/10548408.2015.1075459

[B46] Lee Y.-K. (2016). Impact of government policy and environment quality on visitor loyalty to Taiwan music festivals: moderating effects of revisit reason and occupation type. Tour. Manag. 53, 187–196. 10.1016/j.tourman.2015.10.004

[B47] Lee Y.-K. Lee C.-K. Choi J. Yoon S.-M. Hart R. J. (2014). Tourism's role in urban regeneration: examining the impact of environmental cues on emotion, satisfaction, loyalty, and support for Seoul's revitalized Cheonggyecheon stream district. J. Sustain. Tour. 22, 726–749. 10.1080/09669582.2013.871018

[B48] Lee Y.-K. Lee C.-K. Lee S.-K. Babin B. J. (2008). Festivalscapes and patrons' emotions, satisfaction, and loyalty. J. Bus. Res. 61, 56–64. 10.1016/j.jbusres.2006.05.009

[B49] Leenders M. A. (2010). The relative importance of the brand of music festivals: a customer equity perspective. J. Strateg. Mark. 18, 291–301. 10.1080/09652541003768061

[B50] Leri I. Theodoridis P. (2019). The effects of the winery visitor experience on emotions, satisfaction and on post-visit behaviour intentions. Tour. Rev. 74, 480–502. 10.1108/TR-07-2018-0092

[B51] Li Y. Shang H. (2020). Service quality, perceived value, and citizens' continuous-use intention regarding e-government: empirical evidence from China. Inf. Manage. 57:103197. 10.1016/j.im.2019.103197

[B52] Li Y. N. Wood E. H. (2016). Music festival motivation in China: free the mind. Leis. Stud. 35, 332–351. 10.1080/02614367.2014.962588

[B53] Lin L. Huang Z. Othman B. Luo Y. (2020). Let's make it better: an updated model interpreting international student satisfaction in China based on PLS-SEM approach. PLoS ONE 15:e0233546. 10.1371/journal.pone.0242583 32628675 PMC7337283

[B54] Lin S.-C. Tseng H.-T. Shirazi F. Hajli N. Tsai P.-T. (2023). Exploring factors influencing impulse buying in live streaming shopping: a stimulus-organism-response (SOR) perspective. Asia Pac. J. Mark. Logist. 35, 1383–1403. 10.1108/APJML-12-2021-0903

[B55] Liu M. Xu Y. Hu Y. Li C. Sun F. Chen T. (2014). A century of the evolution of the urban area in Shenyang, China. PLoS ONE 9:e98847. 10.1371/journal.pone.0098847 24893167 PMC4043834

[B56] Lu C. Y. Wang Y. Suhartanto D. (2024). Memory impressions in slow tourism: intrapersonal and interpersonal authenticity as antecedents. Int. J. Tour. Res. 26:e2604. 10.1002/jtr.2604

[B57] Matheson C. M. (2008). Music, emotion and authenticity: a study of Celtic music festival consumers. J. Tour. Cult. Change 6, 57–74. 10.1080/14766820802140448

[B58] Meng B. Luo D. (2024). Family tourists' emotional responses at world heritage sites (WHSs): the effects of existential authenticity and interpersonal interaction. Tour. Rev. 79, 840–854. 10.1108/TR-01-2023-0053

[B59] Miao M. Jalees T. Zaman S. I. Khan S. Hanif N.-A. Javed M. K. (2022). The influence of e-customer satisfaction, e-trust and perceived value on consumer's repurchase intention in B2C e-commerce segment. Asia Pac. J. Mark. Logist. 34, 2184–2206. 10.1108/APJML-03-2021-0221

[B60] Moon H. Han H. (2019). Tourist experience quality and loyalty to an island destination: the moderating impact of destination image. J. Travel Tour. Mark. 36, 43–59. 10.1080/10548408.2018.1494083

[B61] Nunes P. Birdsall C. (2022). Curating the urban music festival: festivalisation, the ‘shuffle' logic, and digitally-shaped music consumption. Eur. J. Cult. Stud. 25, 679–702. 10.1177/13675494211008646

[B62] Nunnally J. C. Bernstein I. H. (1994). Psychometric Theory (3rd Edn.). New York: McGraw-Hill.

[B63] Oliver R. L. (1999). Whence consumer loyalty? Jour. Mark. 63, 33–44. 10.1177/00222429990634s105

[B64] Özdemir C. Düşmezkalender E. Seçilmiş C. Yilmaz V. Yolal M. (2023). Emotion and social identification in music festivals on young's subjective well-being. J. Youth Stud. 27, 851–868. 10.1080/13676261.2023.2174011

[B65] Pakurár M. Haddad H. Nagy J. Popp J. Oláh J. (2019). The service quality dimensions that affect customer satisfaction in the Jordanian banking sector. Sustainability 11:1113. 10.3390/su11041113

[B66] Park E. Choi B.-K. Lee T. J. (2019). The role and dimensions of authenticity in heritage tourism. Tour. Manag. 74, 99–109. 10.1016/j.tourman.2019.03.001

[B67] Perez-Monteagudo A. Curras-Perez R. (2022). Live and online music festivals in the COVID-19 era: analysis of motivational differences and value perceptions. Rev. Bus. Manag. 24, 420–438. 10.7819/rbgn.v24i3.4196

[B68] Perron-Brault A. De Grandpré F. Legoux R. Dantas D. C. (2020). Popular music festivals: an examination of the relationship between festival programs and attendee motivations. Tour. Manag. Perspect. 34:100670. 10.1016/j.tmp.2020.100670

[B69] Pestana M. H. Parreira A. Moutinho L. (2020). Motivations, emotions and satisfaction: the keys to a tourism destination choice. J. Destin. Mark. Manag. 16:100332. 10.1016/j.jdmm.2018.12.006

[B70] Pine B. J. Gilmore J. H. (1998). Welcome to the Experience Economy. Cambridge, MA, USA: Harvard Business Review Press. 10181589

[B71] Qiu H. Wang X. Wu M.-Y. Wei W. Morrison A. M. Kelly C. (2023). The effect of destination source credibility on tourist environmentally responsible behavior: an application of stimulus-organism-response theory. J. Sustain. Tour. 31, 1797–1817. 10.1080/09669582.2022.2067167

[B72] Raab C. Berezan O. Krishen A. S. Tanford S. (2016). What's in a word? Building program loyalty through social media communication. Cornell Hosp. Q. 57, 138–149. 10.1177/1938965515619488

[B73] Saha P. Nath A. Sit K. (2023). Re-examining the roles of experience quality at festivals: a comparative analysis using SEM and fsQCA. Int. J. Contemp. Hosp. Manag. 35, 1802–1823. 10.1108/IJCHM-03-2022-0408

[B74] Sahin A. Kiliçlar A. (2023). The effect of tourists' gastronomic experience on emotional and cognitive evaluation: an application of S-O-R paradigm. J. Hosp. Tour. Insights 6, 595–612. 10.1108/JHTI-09-2021-0253

[B75] Sann R. Luecha P. Rueangchaithanakun R. (2023). The effects of virtual reality travel on satisfaction and visiting intention utilizing an extended stimulus-organism-response theory: perspectives from Thai tourists. J. Hosp. Tour. Insights. 7, 2684–2703. 10.1108/JHTI-05-2023-0321

[B76] Schermelleh-Engel K. Moosbrugger H. Müller H. (2003). Evaluating the fit of structural equation models: tests of significance and descriptive goodness-of-fit measures. Methods Psychol. Res. Online 8, 23–74. 10.23668/psycharchives.12784

[B77] Segars A. H. (1997). Assessing the unidimensionality of measurement: a paradigm and illustration within the context of information systems research. Omega 25, 107–121. 10.1016/S0305-0483(96)00051-5

[B78] Seo Y.-J. Um K.-H. (2023). The role of service quality in fostering different types of perceived value for student blended learning satisfaction. J. Comput. High. Educ. 35, 521–549. 10.1007/s12528-022-09336-z 36033976 PMC9398053

[B79] Sisson A. D. Alcorn M. R. (2022). How was your music festival experience? Impacts on loyalty, word-of-mouth, and sustainability behaviors. Event Manage. 26, 565–585. 10.3727/152599521X16288665119495

[B80] Skandalis A. Banister E. Byrom J. (2024). Spatial authenticity and extraordinary experiences: music festivals and the everyday nature of tourism destinations. J. Travel Res. 63, 357–370. 10.1177/00472875231159054

[B81] Smironva E. Kiatkawsin K. Lee S. K. Kim J. Lee C. H. (2020). Self-selection and non-response biases in customers' hotel ratings–a comparison of online and offline ratings. Curr. Issues Tour. 23, 1191–1204. 10.1080/13683500.2019.1599828

[B82] Su X. Li X. Chen W. Zeng T. (2020). Subjective vitality, authenticity experience, and intangible cultural heritage tourism: an empirical study of the puppet show. J. Travel Tour. Mark. 37, 258–271. 10.1080/10548408.2020.1740141

[B83] Su Y. Xu J. Sotiriadis M. Shen S. (2021). Authenticity, perceived value and loyalty in marine tourism destinations: the case of Zhoushan, Zhejiang Province, China. Sustainability 13:3716. 10.3390/su13073716

[B84] Su Z. Lai S. Zhang M. (2024). Post-pandemic outdoor music festival consumption behaviour intention: an integrative approach. Leis. Stud. 1–21. 10.1080/02614367.2024.2324824

[B85] Suhartanto D. Brien A. Primiana I. Wibisono N. Triyuni N. N. (2020). Tourist loyalty in creative tourism: the role of experience quality, value, satisfaction, and motivation. Curr. Issues Tour. 23, 867–879. 10.1080/13683500.2019.1568400

[B86] Tan K.-L. Ho J.-M. Sim A. K. S. Dubos L. Cham T.-H. (2023). Unlocking the secrets of Miri country music festival in Malaysia: a moderated-mediation model examining the power of FOMO, flow and festival satisfaction in driving revisiting intentions. Asia Pac. J. Tour. Res. 28, 416–432. 10.1080/10941665.2023.2245500

[B87] Tan K.-L. Sim A. K. S. Chai D. Beck L. (2020). Participant well-being and local festivals: the case of the Miri country music festival, Malaysia. Int. J. Event Festiv. Manag. 11, 433–451. 10.1108/IJEFM-02-2020-0007

[B88] Tanford S. (2017). Festival attributes and perceptions: a meta-analysis of relationships with satisfaction and loyalty. Tour. Manag. 61, 209–220. 10.1016/j.tourman.2017.02.005

[B89] Walls A. R. Okumus F. Wang Y. Kwun D. J.-W. (2011). An epistemological view of consumer experiences. Int. J. Hosp. Manag. 30, 10–21. 10.1016/j.ijhm.2010.03.00830912274

[B90] Wang N. (1999). Rethinking authenticity in tourism experience. Ann. Tour. Res. 26, 349–370. 10.1016/S0160-7383(98)00103-036440384

[B91] Wang R. Codina R. Sun Y. Ding X. (2024). Experience, satisfaction and loyalty in the context of online music festivals in China. Int. J. Event Festiv. Manag. 15, 228–248. 10.1108/IJEFM-04-2023-0034

[B92] Weston R. Gore P. A. (2006). A brief guide to structural equation modeling. Couns. Psychol. 34, 719–751. 10.1177/0011000006286345

[B93] Whittaker T. A. Schumacker R. E. (2022). A Beginner's Guide to Structural Equation Modeling. New York: Routledge.

[B94] Wong I. A. Ji M. Liu M. T. (2018). The effect of event supportive service environment and authenticity in the quality–value–satisfaction framework. J. Hosp. Tour. Res. 42, 563–586. 10.1177/1096348015614957

[B95] Wu H.-C. Cheng C.-C. Ai C.-H. (2018). A study of experiential quality, experiential value, trust, corporate reputation, experiential satisfaction and behavioral intentions for cruise tourists: the case of Hong Kong. Tour. Manag. 66, 200–220. 10.1016/j.tourman.2017.12.011

[B96] Wu H.-C. Li T. (2017). A study of experiential quality, perceived value, heritage image, experiential satisfaction, and behavioral intentions for heritage tourists. J. Hosp. Tour. Res. 41, 904–944. 10.1177/1096348014525638

[B97] Yao Y. Zhao X. Ren L. Jia G. (2023). Compensatory travel in the post COVID-19 pandemic era: how does boredom stimulate intentions? J. Hosp. Tour. Manag. 54, 56–64. 10.1016/j.jhtm.2022.12.003

[B98] Yi X. Lin V. S. Jin W. Luo Q. (2017). The authenticity of heritage sites, tourists' quest for existential authenticity, and destination loyalty. J. Travel Res. 56, 1032–1048. 10.1177/0047287516675061

[B99] Yoon Y.-S. Lee J.-S. Lee C.-K. (2010). Measuring festival quality and value affecting visitors' satisfaction and loyalty using a structural approach. Int. J. Hosp. Manag. 29, 335–342. 10.1016/j.ijhm.2009.10.002

[B100] Yu Y. Lang M. Zhao Y. Liu W. Hu B. (2023). Tourist perceived value, tourist satisfaction, and life satisfaction: evidence from Chinese buddhist temple tours. J. Hosp. Tour. Res. 47, 133–152. 10.1177/10963480211015338

[B101] Zhang H. Zhang X. Yang Y. Ma J. (2024). From nature experience to visitors' pro-environmental behavior: the role of perceived restorativeness and well-being. J. Sustain. Tour. 32, 861–882. 10.1080/09669582.2023.2184314

[B102] Zhang S.-N. Li Y.-Q. Liu C.-H. Ruan W.-Q. (2019). How does authenticity enhance flow experience through perceived value and involvement: the moderating roles of innovation and cultural identity. J. Travel Tour. Mark. 36, 710–728. 10.1080/10548408.2019.1625846

[B103] Zhao S. (2023). Why live music matters: implications from streaming music festivals in the Chinese indie music scene. Cult. Sociol. 17, 457–475. 10.1177/17499755221125147

[B104] Zhao Z.-F. Li Z.-W. (2023). Destination authenticity, place attachment and loyalty: evaluating tourist experiences at traditional villages. Curr. Issues Tour. 26, 3887–3902. 10.1080/13683500.2022.2153012

[B105] Zhou M. Yu H. (2022). Exploring how tourist engagement affects destination loyalty: the intermediary role of value and satisfaction. Sustainability 14:1621. 10.3390/su14031621

